# Genetic and Computational Identification of a Conserved Bacterial Metabolic Module

**DOI:** 10.1371/journal.pgen.1000310

**Published:** 2008-12-19

**Authors:** Cara C. Boutte, Balaji S. Srinivasan, Jason A. Flannick, Antal F. Novak, Andrew T. Martens, Serafim Batzoglou, Patrick H. Viollier, Sean Crosson

**Affiliations:** 1 Department of Biochemistry and Molecular Biology, University of Chicago, Chicago, Illinois, United States of America; 2 Department of Statistics, Stanford University, Stanford, California, United States of America; 3 Department of Computer Science, Stanford University, Stanford, California, United States of America; 4 Stanford Genome Technology Center, Stanford University, Palo Alto, California, United States of America; 5 Department of Molecular Biology and Microbiology, Case Western Reserve University, Cleveland, Ohio, United States of America; 6 The Committee on Microbiology, University of Chicago, Chicago, Illinois, United States of America; 7 Graduate Program in Biophysical Sciences, University of Chicago, Chicago, Illinois, United States of America; Massachusetts Institute of Technology, United States of America

## Abstract

We have experimentally and computationally defined a set of genes that form a conserved metabolic module in the α-proteobacterium *Caulobacter crescentus* and used this module to illustrate a schema for the propagation of pathway-level annotation across bacterial genera. Applying comprehensive forward and reverse genetic methods and genome-wide transcriptional analysis, we (1) confirmed the presence of genes involved in catabolism of the abundant environmental sugar *myo*-inositol, (2) defined an operon encoding an ABC-family *myo*-inositol transmembrane transporter, and (3) identified a novel *myo*-inositol regulator protein and *cis*-acting regulatory motif that control expression of genes in this metabolic module. Despite being encoded from non-contiguous loci on the *C. crescentus* chromosome, these *myo*-inositol catabolic enzymes and transporter proteins form a tightly linked functional group in a computationally inferred network of protein associations. Primary sequence comparison was not sufficient to confidently extend annotation of all components of this novel metabolic module to related bacterial genera. Consequently, we implemented the Graemlin multiple-network alignment algorithm to generate cross-species predictions of genes involved in *myo*-inositol transport and catabolism in other α-proteobacteria. Although the chromosomal organization of genes in this functional module varied between species, the upstream regions of genes in this aligned network were enriched for the same palindromic *cis*-regulatory motif identified experimentally in *C. crescentus*. Transposon disruption of the operon encoding the computationally predicted ABC *myo*-inositol transporter of *Sinorhizobium meliloti* abolished growth on *myo*-inositol as the sole carbon source, confirming our cross-genera functional prediction. Thus, we have defined regulatory, transport, and catabolic genes and a *cis*-acting regulatory sequence that form a conserved module required for *myo*-inositol metabolism in select α-proteobacteria. Moreover, this study describes a forward validation of gene-network alignment, and illustrates a strategy for reliably transferring pathway-level annotation across bacterial species.

## Author Summary

More than 1,000 microbial genomes have been sequenced to date, containing millions of predicted genes. While the broad functional category of many of these individual genes can be reliably predicted using sequence homology, sequence information alone is often insufficient to assign a gene a specific cellular function. Closing this gap in our understanding of gene function will require tremendous experimental effort over a broad phylogenetic cross-section of model microbes, along with computational methods for high-confidence extrapolation of functional information from model organisms to other species. Here, we report the experimental identification of a novel genetic module in the model α-proteobacterium *C. crescentus* that controls transport and catabolism of the abundant environmental sugar *myo*-inositol. A combination of computational methods for probabilistic protein-network assignment and gene-network alignment were required to reliably extend the annotation of genes in this metabolic module to related bacterial genera. Our computational predictions of the operon encoding the ABC *myo*-inositol transporter and an essential enzyme for *myo*-inositol catabolism in *S. meliloti* were validated experimentally, demonstrating the feasibility of our method for high-confidence propagation of pathway-level annotation across species.

## Introduction

Inositol, or cyclohexanehexol, is one of the most abundant carbohydrates in freshwater and terrestrial ecosystems [1]. Phosphorylated and lipidated derivatives of inositol serve as important signaling molecules in eukaryotic cells and are critical components of cellular membranes. Among prokaryotes, several species of cyanobacteria, eubacteria and archaea are able to synthesize and derivitize inositol [2]. These molecules serve functional roles as antioxidants, osmolytes, cell membrane components, and as carbon storage substrates [3],[4]. Inositol can also serve as the sole carbon and energy source for many bacterial species [5]–[9] and, in its phosphorylated forms, as a source of phosphorus [10]. Thus inositol is an important biomolecule that is involved in multiple aspects of eukaryotic and prokaryotic cellular physiology and is also a critical nutrient and energy source positioned at the intersection of environmental carbon and phosphorus cycles [1].

**Figure 1:**
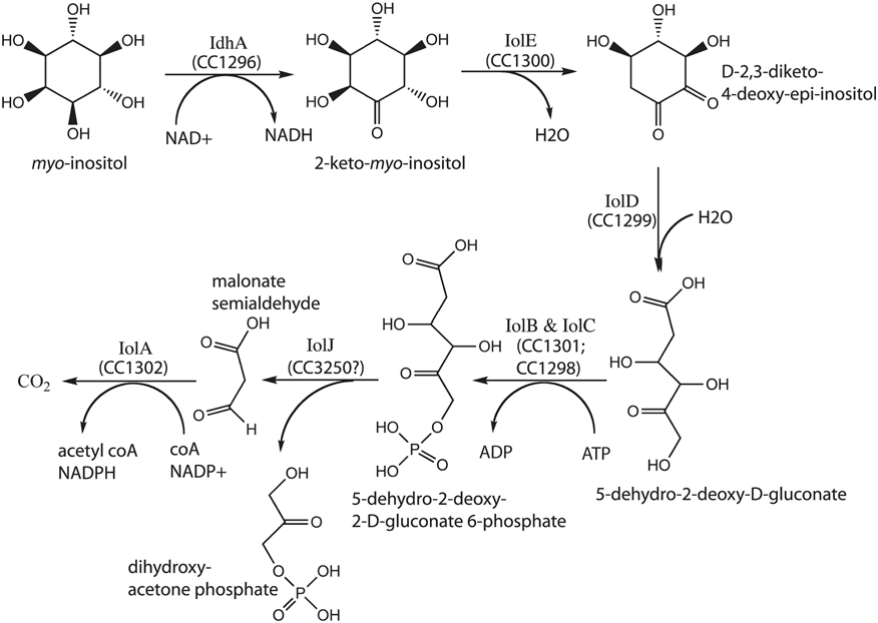
Biochemical pathway of *myo*-inositol degradation in *C. crescentus*. Functional assignments of the catabolic genes are based on biochemical work in *B. subtilis* [14]. All of the genes for this catabolic pathway are present in the *C. crescentus myo*-inositol catabolic locus diagrammed in Figure 2, except the gene *iolJ*. Protein CC3250, annotated as a fructose bisphosphate aldolase, has a high similarity to IolJ of *B. subtilis* (BLAST score<e^−40^). This gene is upregulated 1.9-fold in *myo*-inositol relative to glucose. We propose it is the most likely candidate to carry out the enzymatic function of IolJ in *C. crescentus*.

While cells can derivitize inositol into many different chemical species, the unmodified *myo* form of inositol (*cis*-1,2,3,5-*trans*-4,6-cyclohexanehexol) is among the most abundant species in the environment [1]. The *myo*-inositol degradation pathway has been characterized biochemically in *Klebsiella aerogenes* [5], [11]–[13] and *Bacillus subtilis* [14]. In this pathway, seven proteins convert *myo*-inositol to CO_2_, acetyl CoA and dihydroxy-acetone phosphate (Figure 1). Structural and regulatory genes required for *myo*-inositol catabolism have been identified and characterized in several gram-positive species, including *B. subtilis* [7],[14], *Clostridium perfringens* [8], *Corynebacterium glutamicum* [9], and *Lactobacillus casei* [15], and in the gram-negative bacteria *Rhizobium leguminosarum* bv. viciae [6],[16], *Sinorhizobium meliloti* [3] and *Sinorhizobium fredii* [17]. Gram positives generally exhibit complete and contiguous catabolic operons that are adjacent to genes encoding *myo*-inositol transporters of the major facilitator superfamily; expression of these genes is controlled by transcriptional regulators of the DeoR or LacI families [7]–[9],[18]. Among the gram negatives, genes involved in *myo*-inositol metabolism are more dispersed across the chromosome. This lack of chromosomal co-location and the difficulty in assigning function to transporter and regulatory proteins using sequence homology alone [19],[20] has made comprehensive identification of the *myo*-inositol genetic modules more difficult in these species. In this study we have defined the structural and regulatory components of a genetic module controlling *myo*-inositol transport and catabolism in the gram-negative α-proteobacterium *Caulobacter crescentus*, and reliably extended this experimental functional annotation to other bacterial genera using a combination of computational network prediction and alignment methods.

## Materials and Methods

### Strain Construction and Culture Conditions

*C. crescentus* strain CB15N (NA1000) [21] and strains derived from it were grown in peptone/yeast extract (PYE) or M2 minimal broth [22]. Minimal broth was supplemented with either 0.2% (w/v) *myo*-inositol (M2I) or 0.2% (w/v) glucose (M2G). Directed deletion strains were constructed by ligating approximately 500 base pair regions flanking the 5′ and 3′ regions of the gene to be deleted into the suicide plasmid pNPTS138 (see Table 1) using the *Eco*RI and *Hin*dIII restriction sites. pNPTS138 carries the *npt*I gene to select for single integrants on kanamycin and the *sac*B gene for counterselection on sucrose. The pNPTS138-derived deletion plasmids were transformed into CB15N by electroporation. Initial selection was on 25 µg/ml kanamycin, which was followed by overnight growth in nonselective media and then plating on 3% sucrose to select for cells that had undergone a second crossover event to excise the gene. PCR was used to confirm chromosomal deletions. Cloned fragments to generate pNPTS138 deletion plasmids had the following chromosomal coordinates: *ibpA* upstream = 955,261–956,039; *ibpA* downstream = 956,772–957,359. *iatA* upstream = 956,357–956,940; *iatA* downstream = 958,429–959,014. *iatP* upstream = 957,949–958,539; *iatP* downstream = 959,410–960,005. *iolR* upstream = 1,442,941–1,443,408; *iolR* downstream = 1,444,244–1,444,741. All gene deletions were in-frame. The deletion of *iolR* (CC1297) left the first and last 6 codons intact. The deletion of *ibpA* (CC0859) left the first 45 and last 38 codons intact. The deletion of *iatA* (CC0860) left the first 12 and last 9 codons intact. The deletion of *iatP* (CC0861) left the first 30 and last 13 codons intact. A transporter complementation plasmid was generated by cloning the full transporter locus plus promoter region into the *Kpn*I and *Nde*I sites of the replicating plasmid pMT630 [23]; cloned chromosomal coordinates = 955,261–960,056.

**Table 1:** Strains.

Strain number	Genotype	Reference
FC3	*E. coli* MT607/pRK600	[30]
FC20	*Caulobacter crescentus* CB15N (NA1000)	[21]
FC354	CB15N *ibpA*::*Himar-1*	This study
FC362	CB15N *iolD*:: *Himar-1*	This study
FC536	CB15N *idhA*:: *Himar-1*	This study
FC55	*E. coli* DH10B/pNPTS138	Dickon Alley
FC485	*E. coli* NEB5α/pNPTS138Δ*ibpA*	This study
FC488	CB15NΔ*ibpA*	This study
FC486	NEB5α/pNPTS138Δ*iatA*	This study
FC489	CB15NΔ*iatA*	This study
FC379	*E. coli* TOP10/pNPTS138Δ*iatP*	This study
FC405	CB15NΔ*iatP*	This study
FC341	*E. coli* TOP10F'/pMT630	[23]
FC381	CB15N/pMT630	This study
FC687	CB15NΔ*ibpA*/pMT630-*ibpA-iatA-iatP* operon	This study
FC688	CB15NΔ*iatA*/ pMT630-*ibpA-iatA-iatP* operon	This study
FC689	CB15NΔ*iatP*/ pMT630-*ibpA-iatA-iatP* operon	This study
FC457	CB15NΔ*iolR*	This study
FC415	TOP10/pRKLac290-*iolC* promoter	This study
FC418	CB15N/pRKLac290-*iolC* promoter	This study
FC470	CB15NΔ*iolR*/pRKLac290-*iolC* promoter	This study
FC558	CB15N/pRKLac290-m1 mutant *iolC* promoter	This study
FC571	CB15N/pRKLac290-m2 mutant *iolC* promoter	This study
FC416	TOP10/pRKLac290-*idhA* promoter	This study
FC420	CB15N/pRKLac290-*idhA* promoter	This study
FC471	CB15NΔ*iolR*/pRKLac290-*idhA* promoter	This study
FC521	TOP10/pRKLac290-*ibpA* promoter	This study
FC524	CB15N/pRKLac290-*ibpA* promoter	This study
FC525	CB15NΔ*iolR*/pRKLac290-*ibpA* promoter	This study
FC692	*Sinorhizobium meliloti* Rm2011	[24]
FC693	Rm2011 *iolA*::Tn*5*	[24]
FC694	Rm2011 *ibpA*::Tn*5*	[24]

*Sinorhizobium meliloti* Rm2011 and strains derived from it were obtained from the lab of Anke Becker (Bielefeld University, Germany) [24]. *S. meliloti* was grown in either LB or GTS minimal medium [25] supplemented with 0.2% glucose (GTS-G) or 0.2% inositol (GTS-I) as the sole carbon source. All strains used in this study are listed in Table 1.

### Transposon Screen for *C. crescentus* Strains Deficient in Growth on *myo*-Inositol

A library of ≈16,000 individual *C. crescentus* CB15N mutant strains carrying either the *Mariner*–based *Himar-1* transposon [26], the *Mu*-based Hyper*Mu* transposon (Epicentre, Madison, WI), or the Tn*5*-derived EZ-Tn*5* transposon (Epicentre, Madison, WI) was generated and stored in 96-well format (Pritchard, Matteson and Viollier, unpublished). This transposon library was replica stamped from the 96-well plates onto M2 agar supplemented with either 0.2% (w/v) *myo*-inositol (M2I), 0.1% cellobiose (M2C) or 0.2% glucose (M2G). Strains that grew on M2C and M2G, but not M2I were considered to have inositol-conditional mutations. Mapping of the transposon insertion site in *C. crescentus Himar-1*, Hyper *Mu*, and Ez-Tn*5* mutant strains deficient for growth on M2I was determined by isolating chromosomal DNA, digesting with *Hin*PI for 10 minutes at 37°C, ligating the digested genomic fragments into circles using T4 DNA ligase, and transforming 1 µl of this ligation reaction into electrocompetent *E. coli* EC100D *pir-116* cells (Epicentre, Madison, WI). These transposons all carry an R6K origin that replicates in a *pir* ^+^ strain of *E. coli*. The circularized transposon plasmids were then isolated from *E. coli* and silica-column purified (Novagen, Madison, WI). The location of the transposon insertion was determined via a single primer sequencing extension reaction from the purified, circularized transposon plasmids. The oligos used to map these transposons are as follows: *Himar-1*-GATATTGCTGAAGAGCTTGGCGGCGAA; Ez-Tn*5*- CTACCCTGTGGAACACCTACATCT; Hyper-*Mu*-AGAGATTTTGAGACAGGATCCG.

### DNA Microarray Analysis of Genes Regulated by Growth on *myo*-Inositol Relative to Glucose

Wild type *C. crescentus* CB15N cells were grown in either M2 minimal medium supplemented with 0.2% (w/v) glucose (M2G) or M2 supplemented with 0.2% *myo*-inositol (M2I) to OD_660_ of 0.3–0.4. 5 ml of 4 replicate cultures (for each carbon condition) were spun down at 10,000× g for 30 seconds, the supernatant was removed, and the cell pellets were flash frozen in liquid nitrogen. RNA was isolated from these cells by incubating in 1 ml of Trizol (Invitrogen, Carlsbad, CA) at 65°C for 10 minutes, adding chloroform, vortexing, spinning, and extracting the aqueous layer. Nucleic acid in the aqueous layer was isopropanol precipitated overnight at −80°C followed by a 30 minute centrifugation at 16,000× g. The ethanol-washed and air-dried nucleic acid pellet was resuspended in 50 µl of nuclease-free water (IDT, Coralville, IA). 1 µl of RNase-free DNase I (Ambion, Austin, TX) was added to the sample and incubated at room temperature for two hours to remove any residual DNA. The nucleic acid in this digested sample was then acid phenol-chloroform (Ambion, Austin, TX) extracted, ethanol precipitated at −80°C overnight, and centrifuged at 16,000× g to produce a DNA-free RNA pellet. RNA quality was assessed via agarose gel electrophoresis and RNA concentration determined by UV spectrophotometry using a Shimadzu UV-1650 (Kyoto, Japan).

Labeled indodicarbocyanine-dCTP (Cy3) and indocarbocyanine-dCTP (Cy5) cDNA was generated from 20 µg of total RNA by reverse transcription with Superscript II reverse transcriptase (Invitrogen, Carlsbad, CA) using 1 µg of random hexamer primers (Invitrogen, Carlsbad, CA). 2 samples of cDNA from each RNA type (M2I and M2G) were Cy3 labeled and 2 were Cy5 labeled. Dye-swapped cDNA from the remaining two samples was generated in order to minimize dye bias in the microarray analysis. Paired Cy3 and Cy5 labeled cDNA from the M2G and M2I samples were hybridized onto spotted DNA oligo arrays using a protocol previously described [27]. After hybridization and washing, the arrays were scanned with a GenePix 4000B scanner (Axon Instruments). Scanned spots were converted to ratios (red/green) with GenePix Pro 5.0 software. Expression ratio data (glucose/inositol) for the four biological replicates were normalized by median centering and analyzed using the Significance Analysis for Microarrays (SAM) package [28]. Genes that showed a 2-fold or greater mean expression change (either up or down in *myo*-inositol relative to glucose) and that were determined to be significant in SAM using a 5% false discovery cutoff are included in Table S1. DNA microarray data have been deposited in the Gene Expression Omnibus (GEO) database (http://ncbi.nlm.nih.gov/geo) under accession number GSE12414.

### Promoter Activity Assays

To measure the promoter activities of inositol-regulated genes/operons, we first PCR-amplified promoter regions of three genes. The *idhA* promoter region extends from chromosomal coordinate 1,443,231 to 1,443,769; the *iolC* promoter region extends from coordinate 1,443,840 to 1,444,387; the *ibpA* promoter extends from coordinate 955,627 to 955,895. These fragments were digested and cloned into the reporter plasmid pRKlac290 [29] using the *Eco*RI and *Hin*dIII sites. The resulting promoter-*lacZ* transcriptional fusion plasmids were introduced into *C. crescentus* CB15N or CB15NΔ*iolR* by tri-parental conjugation using the *E. coli* helper strain FC3, which carries the pRK600 plasmid [30].

β-galactosidase activity from the LacZ-promoter reporter strains was determined colorimetrically using cells in log phase (0.1–0.3 OD_660_) at 30°C; Z-buffer (60 mM Na_2_HPO_4_, 60 mM NaH_2_PO_4_, 10 mM KCl, 1 mM MgSO_4_) and an excess of o-nitrophenyl-β-D-galactopyranoside was added to chloroform-permeabilized cells and absorbance was measured at 420 nm on a Spectronic Genesys 20 Spectrophotometer (ThermoFisher Scientific, Waltham, MA). Two palindromic consensus motifs in the *iolC* promoter - that we also identified upstream of several *myo*-inositol-regulated genes - were mutated by PCR using mismatched oligos. The site-directed mutagenesis PCR was followed by 1 hour of *Dpn*I digestion, and 1 µL of the digested reaction was transformed into electrocompetent *E. coli* TOP10 cells (Invitrogen, Carlsbad, CA). pCR-BluntII plasmids (Invitrogen, Carlsbad, CA) containing the mutant *iolC* promoters were amplified and purified, and the mutated *iolC* promoters were excised with *Eco*RI and *Hin*dIII, and sub-cloned into pRKLac290 to generate mutated P_i*olC*_-*lacZ* transcriptional fusions. The motif that is positioned between 104 and 119 bases upstream of the predicted start codon of *iolC* was mutated from TG**G**ACCATATGT**T**C**C**A to TG**T**ACCATATGT**A**C**A**A. The motif positioned between 45 and 60 bases upstream of the predicted *iolC* start codon was mutated from T**G**G**A**ATATGCG**T**TACA to T**T**G**C**ATATGCG**G**TACA.

### Growth Curves

Cell growth in different media types was measured in triplicate in bulk culture grown in 13 mm glass tubes in an Infors tube shaker (ATR Biotech, Laurel, MD), at 30°C, 220 rpm. Density measurements for individual cultures were taken hourly up to 0.3 OD_660_ in a Genesys20 Spectrophotometer (ThermoFisher Scientific, Waltham, MA) and the growth rate was determined by fitting the data to an exponential growth equation:
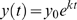
in Prism (GraphPad Software, San Diego) , where *y_0_* is the initial cell density, *k* is growth rate, and *t* is time.

### Computational Protein Network Integration

For each of 305 sequenced prokaryotic genomes, we assembled a battery of different predictors of protein association including coexpression, coinheritance, colocation, and coevolution. We formulated the network integration problem as a binary classifier, where the goal is to distinguish functionally linked protein pairs (L = 1) from non-interacting pairs (L = 0). In this formulation, a vector of interaction predictors is the input to a binary classifier function, which returns the integrated probability that two proteins are functionally linked. To calculate the mapping between raw interaction data and integrated probabilities, the classifier function is trained on a set of known interactions. Applying this classifier to predict interaction probabilities for all protein pairs in a genome yields a probabilistic protein interaction network.

Specifically, we generated the training set of known interactions by using KEGG [31] classifications of individual proteins to produce an annotation of protein pairs. For each pair we recorded if the proteins had overlapping annotations (L = 1), if both were in entirely non-overlapping KEGG categories (L = 0), or if either protein lacked an annotation code or was marked as unknown (L = ?). We also calculated four functional genomic and experimental predictors: *1)* coexpression; the Pearson correlation between genes in publically-available DNA microarray expression data, *2)* coinheritance; the Pearson correlation between protein phylogenetic profiles [32], *3)* coevolution; the Pearson correlation between protein distance matrices, taken elementwise [33], and *4)* collocation; the average chromosomal distance between ORFs. Each of these predictors is defined on a pair of proteins rather than an individual protein and can be arranged in a four dimensional vector:
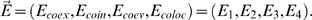
It can be shown empirically that the distribution of functionally linked protein pairs is shifted relative to the distribution of functionally unlinked pairs [34]. Intuitively, this means that each genomic evidence type is a predictor of protein functional interaction. We can combine these predictors to obtain the integrated probability of protein interaction via Bayes' rule [35].

In practice, the quotient formula for the Bayesian posterior probability is quite sensitive to fluctuations in the denominator. To deal with this, we used bootstrap aggregation [36] to smooth the posterior as follows:
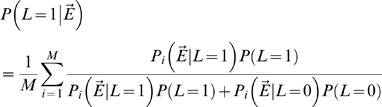
where M is the number of bootstrap replicates.

Thus, for each pair of proteins, we have a value P(L = 1|E_1_,E_2_,E_3_,E_4_) which represents the integrated probability of protein interaction over several data types.

Additional computational details underlying this protein network prediction strategy are discussed in Srinivasan, et al. [34]. A web interface for this functional networking database containing predicted networks for 305 bacterial species is available at http://networks.stanford.edu.

### Multiple Network Alignment

Network alignment is a systems-biology analog of sequence alignment that compares protein association networks between different species in an effort to identify conserved functional modules. Such modules are sets of proteins that have both conserved primary sequences and conserved pairwise statistical associations between species. For automated network alignment, we used the experimentally- and computationally-defined *myo*-inositol network from *C. crescentus* as a query module. This module was used to conduct query-to-network alignment searches across computationally-predicted protein interaction networks of 5 related α-proteobacterial species [34]; these interaction networks had been previously defined using the statistical protein network prediction strategy outlined above. The bacterial species included in this alignment were *Sinorhizobium meliloti*, *Mesorhizobium loti*, *Brucella melitensis*, *Agrobacterium tumefaciens*, and *Bradyrhizobium japonicum*. Initial alignment identified the best match to the query in each protein interaction network.

Specifically, we used the Graemlin algorithm [37] to perform automated cross-species alignment. Graemlin incorporates ideas from sequence alignment to perform query-to-network alignment accurately and efficiently. To search multiple networks for matches to a query module, Graemlin first aligns the query module to the evolutionarily closest network by identifying a high scoring pair of proteins within the query and network and aligning them. Then, Graemlin extends the alignment by aligning the pair of proteins that will increase the score of the alignment the most, continuing until it cannot further increase the score of the alignment. The score for aligning a pair of proteins is higher when the proteins are *1)* sequence similar and *2)* connected to many proteins in the current alignment. Once Graemlin aligns the query module to the evolutionarily closest (i.e. highest scoring) network, it aligns the resulting alignment to the next evolutionarily closest network. To perform this alignment it uses the same algorithm that it uses to perform the first alignment, with an adjusted scoring function [37]. Graemlin continues performing alignments in this fashion until it has aligned the query to every network. To date, Graemlin is the only algorithm capable of aligning a query module to more than three networks. Our benchmarks have shown that when aligning a query module to a single network, this method of alignment is more accurate and efficient than existing network alignment algorithms [37].

To improve the predictive power of the alignment, we manually refined the alignment to keep the best candidates in each species using the following criteria: *1)* in each species, we considered only transporter operon candidates in which the three ABC transporter components were contiguous on the chromosome; this resulted in several candidate conserved operons in each species, *2)* in each species, we assessed the similarity of each candidate operon to those in all other species in the alignment. We then calculated, for each protein in the candidate operon, the average BLAST significance score to its predicted counterpart in all other species; the candidate operon with the best average significance score (i.e. lowest average p-value) was selected for inclusion in the final cross-species module. Additional computational details underlying this protein network prediction strategy are discussed in Flannick et al. [37]. The network alignment tool Graemlin 2.0 is available under the GNU public license at http://graemlin.stanford.edu.

### Motif Discovery

We used MEME [38] to locate putative regulatory motifs in the upstream regions of genes in the *C. crescentus myo*-inositol module. In order to refine this motif, and also to investigate its conservation in other species, we used MEME to search 250 base pairs upstream of the predicted translation start sites of genes in the predicted inositol modules in each of the species present in our multi-species network alignment. The MEME search parameters were as follows: motif distribution, 0–1 per sequence; minimum motif width, 6; maximum motif width, 50.

## Results/Discussion

### Forward and Reverse genetic Identification of Genes That Are Essential for Growth on *myo*-Inositol

**Figure 2:**
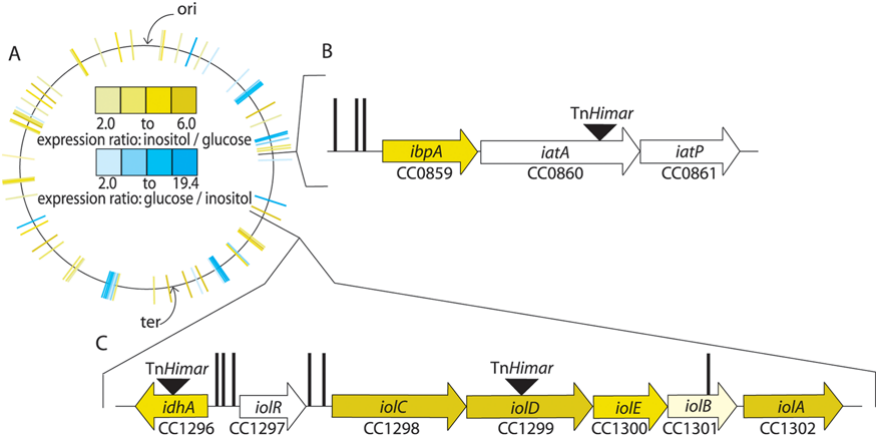
Genomic organization of the *C. crescentus myo*-inositol module and regulation of gene expression by *myo*-inositol and glucose. (A) Diagram of the *C. crescentus* chromosome with microarray expression data overlayed. Colored lines represent genes that are significantly up- (yellow) or down-regulated (blue) during growth on *myo*-inositol relative to glucose as the sole carbon source. The complete set of genes that are significantly up- or down-regulated in *myo*-inositol versus glucose can be found in Table S1. (B) Chromosomal organization of the *myo*-inositol transport operon, and (C) the *myo*-inositol degradation operon. Right-facing arrows indicate genes are on the plus strand of the chromosome; left-facing arrows indicate genes are on the minus strand. The color of the genes corresponds to their degree of regulation in *myo*-inositol relative to glucose (see color scale in the center of panel A). Black triangles represent locations of *Himar* transposon insertions identified in a forward screen for *C. crescentus* strains that cannot utilize *myo*-inositol as the sole carbon source. Vertical black lines indicate the location of the *cis*-acting regulatory motif GGAA-N6-TTCC (see Figure 4).

Using an arrayed library of ≈16,000 mutant *C. crescentus* strains carrying transposon insertions, we conducted a forward genetic screen for mutants that could not grow on *myo*-inositol as the sole carbon source. Three strains, FC354, FC362 and FC536, were discovered that were unable to grow on M2-*myo*-inositol medium (M2I) but exhibited normal growth on PYE, M2-cellobiose (M2C) and M2-glucose (M2G). Strain FC536 has a transposon insertion in the *myo*-inositol 2-dehydrogenase (*idhA*; *CC1296*, NP_420109) gene. The IdhA homolog from *B. subtilis* has been characterized biochemically [39], and is known to catalyze the first dehydrogenation reaction in the *myo*-inositol degradation pathway (Figure 1). Strain FC362 contains a transposon insertion in the *iolD* gene (*CC1299*, NP_420112). IolD has also been characterized in *B. subtilis* where it was shown to catalyze hydrolysis and ring opening of the catabolic intermediate D-2,3-diketo-4-deoxy-epi-inositol to form 5-dehydro-2-deoxy-D-gluconate [14]. The transposon insertion in strain FC362 likely disrupts expression of not only *iolD*, but also genes downstream of *iolD* in the operon encoding other known *myo*-inositol catabolic enzymes (Figure 2C and Figure 1).

The third strain identified in our screen, FC354, contained a transposon that mapped to *CC0860*, a gene encoding a ATPase protein in an operon predicted to encode an ATP-binding cassette (ABC) sugar transporter (Figure 2B). This transporter operon is physically separated on the chromosome from the genes encoding the catabolic enzymes by ≈500 kilobases (Figure 2). ABC sugar transporters are inner-membrane transporters that employ three components - a periplasmic sugar binding protein, a transmembrane permease and a cytoplasmic ATPase - to move sugars from the periplasm to the cytoplasm [40]. To confirm that this transporter operon, *CC0859–CC0861*, is required for growth on *myo*-inositol, we constructed strains with in-frame deletions of each of these genes individually: *C. crescentus* strains CB15NΔ*ibpA* (*CC0859*, inositol binding protein, NP_419676), CB15NΔ*iatA* (*CC0860*, inositol ABC transporter ATPase, NP_419677), and CB15NΔ*iatP* (*CC0861*, inositol ABC transporter permease, NP_419678). Individual in-frame deletions of each of these genes abolished growth on defined medium containing *myo*-inositol as the sole carbon source, but not on defined minimal glucose medium (Table 2) or PYE complex medium. Growth on *myo*-inositol in the individual in-frame transporter deletion strains was restored by complementation with a replicating vector carrying the entire *ibpA-iatA-iatP* locus under the control of its own promoter (Table 2).

**Table 2:** Doubling times, in minutes, of *C. crescentus* strains in M2 minimal medium with either glucose (M2G) or *myo*-inositol (M2I) as the sole carbon source.

Strain	M2-glucose	M2-inositol
CB15N	120.2±1.8	564.4±2.6
CB15N *iolD*::TnMariner	122.0±3.0	No growth
CB15N *iatA*::TnMariner	120.5±1.8	No growth
CB15N *idhA*::TnMariner	121.4±0.1	No growth
CB15NΔ*ibpA*	122.4±1.1	No growth
CB15NΔ*iatA*	121.3±0.9	No growth
CB15NΔ*iatP*	119.3±1.8	No growth
CB15NΔ*ibpA*/pMT630-*iat* operon	156.4±1.3	605.5±4.7
CB15NΔ*iatA*/pMT630-*iat* operon	153.1±0.9	612.7±8.5
CB15NΔ*iatP*/pMT630-*iat* operon	159.3±4.0	625.1±5.0
CB15N / pMT630	157.5±5.4	624.3±6.2

No growth indicates a lack of any visible cell growth after 1 week of shaken incubation.

The inability of *C. crescentus* strains lacking any gene in the *ibpA-iatA-iatP* operon to grow in *myo*-inositol demonstrates that this operon encodes the only inner-membrane *myo*-inositol transporter in *C. crescentus*.

### Microarray Identification of Genes That Are Differentially Expressed in *myo*-Inositol Relative to Glucose

Whole-genome transcriptional profiling using DNA microarrays was conducted to identify genes with differential regulation in *myo*-inositol relative to glucose as the sole carbon source. 50 genes were found to have transcript levels that were at least 2-fold higher in cells grown in *myo*-inositol than in glucose (see Materials and Methods for data analysis parameters) (Figure 2A and Table S1). Among these genes, as expected, are the catabolic genes *idhA* and the *iolECBDA* operon, as well as the gene, *ibpA*, encloding the periplasmic binding protein of the *myo*-inositol ABC transporter.

The most highly induced gene in *myo*-inositol relative to glucose (>4-fold) is isocitrate lysase (CC1764, NP_420572) which catalyzes formation of glyoxylate and succinate from isocitrate. This result suggests that growth of *C. crescentus* on *myo*-inositol shifts energy metabolism toward the glyoxylate cycle relative to growth on glucose. The ATPase subunit of a HlyB-family ABC-transporter (gene *CC1314*, NP_420127) is also four-fold more abundant in *myo*-inositol than in glucose (Table S1). As discussed above, cells with mutations in the *ibpA-iatA-iatP* transporter operon fail to grow on *myo*-inositol as the sole carbon source after one week of incubation (Table 2) providing evidence that this HlyB-family transporter is not a redundant *myo*-inositol transporter. However, this transporter may be involved in transporting derivatized versions of inositol (e.g. inositol phosphates or lipidated inositols).

### Expression of Genes in the *myo*-Inositol Module Is Regulated by IolR and a Conserved *cis*-Acting Sequence

**Figure 3:**
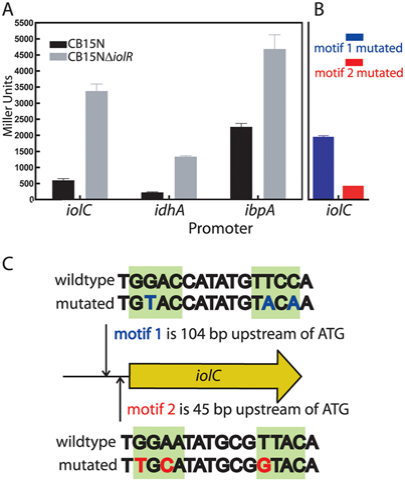
Regulation of genes in the *myo*-inositol module by the transcriptional regulator *iolR* and a conserved promoter sequence. (A) β-galactosidase assays of the *idhA*, *ibpA* and *iolC* promoters in the wild-type and Δ*iolR* backgrounds. (B) β-galactosidase assays of motif 1 (m1) and motif 2 (m2) mutated versions of the *iolC* promoter assayed in a wild-type background. The scale on the Y axis is the same for A and B. All β-galactosidase assays were conducted in PYE complex medium. (C) Schematic of the *iolC* promoter showing the location of the two conserved motifs; the wild-type and mutated versions of motif 1 are shown above the motif location map; wild-type and mutated versions of motif 2 are shown below.

The gene *CC1297* (NP_420110) is annotated as an RpiR-family transcriptional regulator and encodes a putative SIS (Sugar ISomerase; Pfam 01380) domain at its N-terminus. Based on its predicted function as a sugar-binding transcription factor and its chromosomal location adjacent to the *iol* catabolic operon (Figure 2C), we predicted that CC1297 would regulate transcription of the *iol* genes. To test this hypothesis, we constructed a strain with an in-frame deletion of this gene and measured expression from the *idhA*, *iolC* (NP_420111), and *ibpA* promoters in wild type and *CC1297* deletion strains using promoter-*lacZ* fusions as transcriptional reporters. These assays revealed significant derepression of transcription from the *idhA*, *ibpA* and *iolC* promoters in a *CC1297* deletion background when cells were grown in PYE complex medium (Student's t-test; p<0.0001) (Figure 3A). This result is consistent with the idea that CC1297 is a transcriptional regulator of the *iol* genes. As such, we have named this gene *iolR*. Notably, *C. crescentus* IolR is not homologous to the IolR proteins previously described in *Bacillus subtilis*, *Corynebacterium glutamicum* or *Clostridium perfringens* [8],[9],[18] and thus defines a new class of *myo*-inositol regulator proteins. In contrast with these unrelated *myo*-inositol regulator genes, which are induced by *myo*-inositol [7],[9], expression of *C. crescentus iolR* is not regulated by *myo*-inositol based on our microarray transcriptional profiling data.

**Figure 4:**
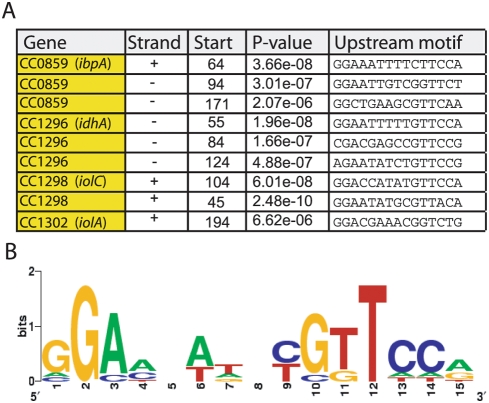
Identification of a conserved motif in the promoters of genes regulated by myo-inositol. (A) The upstream regions of the genes and operons that were shown to be required for growth on *myo*-inositol were searched for common sequence motifs using MEME [38]. This search identified a conserved palindromic motif (MEME e value = 4.4 e^−04^). (B) A weblogo cartoon [44] showing the relative nucleotide frequency at each of the 15 positions in the promoter motif, where frequency is proportional to the height of the letter.

We then sought to identify possible regulatory motifs in the predicted promoter regions of genes in the *myo*-inositol metabolic module of *C. crescentus*. A MEME search [38] of the DNA sequence of these promoters suggested a consensus palindromic motif, **GGAA**NATNCG**TTCC**A that is present upstream of *ibpA*, *idhA*, *iolC* and *iolA* (NP_420115 ) (Figures 2B and C & Figure 4). The *iolC* promoter contains two copies of this motif with MEME e-values less than 10^−8^ (Figure 4) and with good conservation of the palindrome. Motif 1 is 104 bp upstream of the predicted translation start site of *iolC*, while motif 2 is 45 bp upstream (Figure 3C). We mutated each of these two motifs away from consensus (Figure 3C), and measured expression from these mutant *iolC* promoters in complex medium (PYE). Mutation of motif 1 results in significantly higher LacZ activity than the wild-type promoter (Student's t-test; p<0.001), demonstrating that motif 1 is involved in basal repression of *iolC* expression. Mutation of motif 2 does not affect measured promoter activity in PYE (p>0.05) (Figure 3B). These results demonstrate that, in the case of motif 1, the palindromic sequence we have identified in the promoters of genes required for *myo*-inositol metabolism is a functionally relevant regulator of gene expression. Future analysis of the regulatory role of IolR, of motif 2 in the *iolC* promoter, and of the palindromic motifs in the *idhA* and *ibpA* promoters promises to provide insight into additional layers of regulation in this genetic module.

### Computational Prediction of a *myo*-Inositol Catabolic/Transport Module in *Caulobacter crescentus*

**Figure 5:**
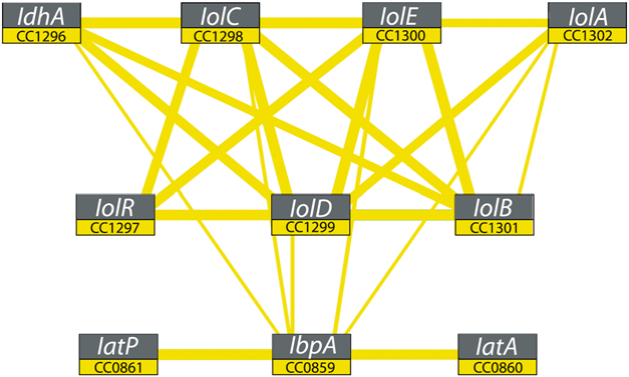
Computational prediction of the *myo*-inositol module in *C. crescentus*. A probabilistic network of protein associations constructed from coexpression, coinheritance, colocation, and coevolution data from *C. crescentus* [34] predicts that genes at two disjoint chromosomal loci (CC0859–CC0861 and CC1296–CC1302) are functionally associated (i.e. these genes are predicted to be part of the same KEGG pathway [45] or GO process [46]). Genes are shown as nodes and associations as edges, with edge widths denoting the strength of association (confidence interval for narrow lines is 30–60%; confidence for thick lines is >60%).

Independent of our experimental work, we applied statistical methods that we previously developed to predict functional associations between genes in prokaryotic genomes (see Materials and Methods and [34]). Figure 5 shows the computationally-predicted “*myo*-inositol module” of *C. crescentus*. This is a subset of our whole-genome *C. crescentus* integrated protein association network, containing proteins encoded in operons at just two distinct chromosomal loci. The first chromosomal locus contains genes (*C. crescentus* gene numbers *CC1296*; *CC1298–CC1302*) that are predicted to be involved in catabolism of *myo*-inositol by sequence homology to known enzymes involved in *myo*-inositol catabolism [5], [11]–[13]. The second locus is an operon containing genes (*CC0859–CC0861*) that are predicted to encode the three components of a canonical ABC transmembrane sugar transporter [41]: a periplasmic sugar-binding protein, an ATPase subunit, and a transmembrane permease. However, the periplasmic sugar-binding protein of this transporter is only generally annotated as a member of the XylF superfamily in the Conserved Domain Database (CDD score<e^−15^) [42], and its true substrate was not known at the time we constructed our microbial protein association networks. The *C. crescentus* inositol module also contains the gene, *CC1297 (iolR)*, which is colocated with the predicted *myo*-inositol catabolic genes and is annotated as encoding a transcriptional regulator.

We found that the transporter and catabolic proteins have strong intra-operon linkage (>80% confidence), which is largely due to high colocation and coinheritance scores (Figure 5). The inter-operon association between the transporter, catabolic, and regulatory proteins, which are encoded from genes at two disparate chromosomal loci, primarily arises from moderate statistical correlations contained within the microarray coexpression component of our model. Using a 30% confidence cutoff, we deduce that the periplasmic sugar-binding protein CC0859 (IbpA) is functionally linked to several genes in the predicted *myo*-inositol catabolic operon (Figure 5). No other transmembrane transporters in the *C. crescentus* genome are predicted to associate with the *myo*-inositol catabolic genes in our network. This linkage between the *myo*-inositol catabolic proteins and the ABC sugar transporter is missed using a single association metric such as colocation, coinheritance or coexpression alone. An integrative statistical model, which incorporates multiple predictors of association, is required to identify this association.

As discussed above, genetic and molecular experiments have confirmed the computationally-predicted association between the ABC sugar transporter and the *myo*-inositol catabolic genes. Specifically, we have shown that 1) proteins encoded by the transporter operon *CC0859–CC0861* (now annotated as IbpA, IatA, and IatP) function to form the sole *myo*-inositol inner-membrane transporter in *C. crescentus*, 2) transposon disruption of the predicted catabolic locus encompassing *CC1296*; *CC1298–CC1302* (annotated as IdhA, IolC, IolD, IolE, IolB, IolA) abolishes growth of *C. crescentus* on *myo*-inositol as the sole carbon source, 3) the transcriptional regulator gene *CC1297* (annotated as IolR) functions to regulate expression of the *myo*-inositol transporter and catabolic genes.

### Computational Prediction of Functionally Homologous *myo*-Inositol Modules in Other Species

**Figure 6:**
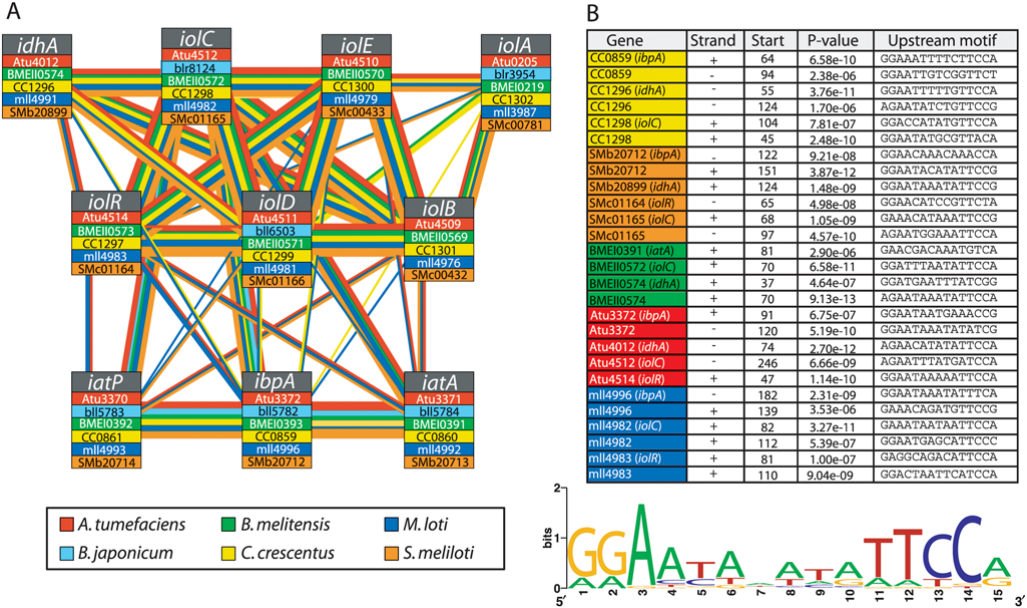
Cross-species module prediction in five other α-proteobacteria. (A) A multiple network alignment constructed using the Graemlin algorithm [37] shows extensive conservation of the *myo*-inositol module across four of five related α-proteobacterial species (*Sinorhizobium meliloti*, *Mesorhizobium loti*, *Agrobacterium tumefaciens*, and *Brucella melitensis*) at both the protein level and the level of inter-protein association. Here, nodes represent groups of sequence-homologous proteins; individual blocks within a node correspond to specific proteins and are color coded by species (see color key). Edges between nodes are likewise color coded by species, with edge widths representing association strength (confidence interval for narrow lines is 30–60%; confidence for thick lines is >60%). (B) Network alignment boosts the signal for promoter motif finding (MEME e value = 4.3 e^−75^). The same palindromic motif is found in the upstream regions of *iolC*, *idhA*, and in the promoter region of the *ibpA-iatA-iatP* operon in all species except *B. japonicum*, in which the *myo*-inositol module is less conserved. A weblogo cartoon [44] is shown below the aligned sequences, where the relative nucleotide frequency at each of the 15 positions in the promoter motif is proportional to the height of the letter.

Using automated cross-species alignment in combination with manual post-refinement (see Materials and Methods) we identified genetic networks in other bacterial species that we predicted to be functionally homologous to the *C. crescentus myo*-inositol network (Figure 6). The cross-species alignment conducted in this study indicates significant conservation of the catabolic, regulatory, and transporter proteins across the six α-proteobacterial species aligned. In addition, there are conserved cross-protein functional linkages within each of these species (Figure 6A). Linkage between the transporter and catabolic proteins is particularly strong in *M. loti* and *S. meliloti* as evidenced by the large number of association edges between transporter, catabolic, and regulatory genes in these species (Figure 6A). The module is least conserved in *B. japonicum*, which is discussed further below.

**Figure 7:**
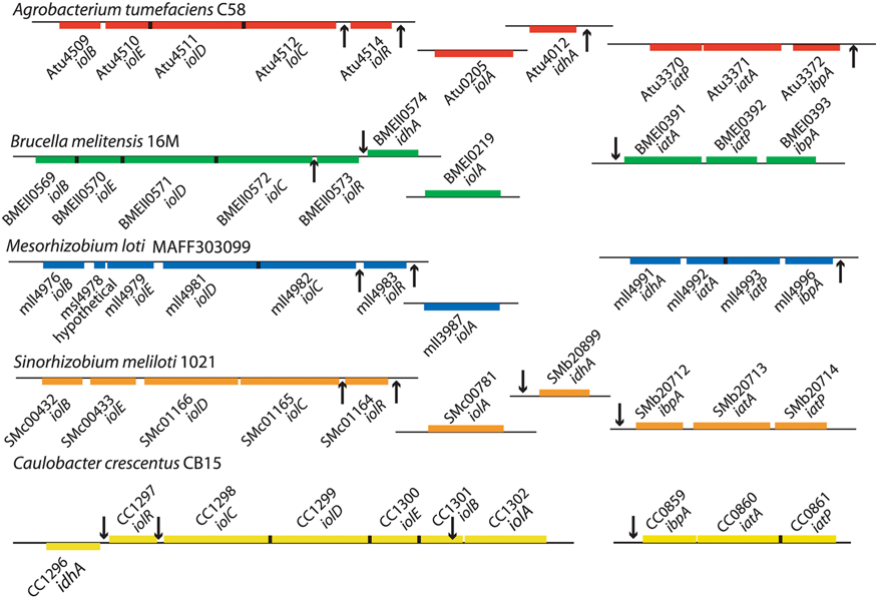
Genomic organization of the conserved *myo*-inositol module in five α-proteobacteria. Of the six species analyzed, the *myo*-inositol metabolic module is most compact in *C. crescentus* (yellow), where is distributed between only two chromosomal loci. *A. tumefaciens* (red) and *S. meliloti* (orange) exhibit homologous chromosomal organization of the module, distributed at four chromosomal sites. *B. melitensis* (green) and *M. loti* (blue) have predicted module components at three sites, although the organization is different: in *M. loti*, *idhA* is transcribed as part of the transporter operon, while *idhA* in *B. melitensis* is adjacent to *iolR* but transcribed off the opposite strand. Genes are shown as colored boxes on a horizontal line. Boxes above the line are genes on the plus strand; boxes below the horizontal line are on the minus strand. Vertical black arrows indicate the location of the cis-acting regulatory motif GGAA-N6-TTCC (see Figure 4 and Figure 6B). Cases where we have identified more than one of these motifs in a particular promoter are only marked with a single arrow.

As discussed above, we discovered a palindromic motif (GGAA-N6-TTCC) with a moderate MEME e-value upstream of several genes in the *C. crescentus* inositol module (Figure 4). By reasoning that conservation at the gene system level may imply conservation at the level of gene regulation, we searched 250 bases upstream of the predicted translational start sites of genes in our cross-species network alignment for sequences related to the palindromic motif identified in *C. crescentus* (Figure 4). In these related sequences, we found 21 more examples of this same motif, which was particularly enriched in the predicted upstream homologs of *iolC*, *idhA*, and the *myo*-inositol ABC transporter operons in these species (Figure 6B and 7). Incorporating the upstream sequences from all species in the Graemlin alignment in a MEME motif search dramatically improved the significance score for this regulatory motif (Figure 6B).

Notably, *B. japonicum* is the only one of the six species in our multiple network alignment in which we could not identify this motif upstream of predicted inositol catabolic and transporter genes. Although it contains strong associations at the transporter nodes and for a number of the metabolic genes, it is missing several other *myo*-inositol catabolic genes and also does not encode a homolog of the regulatory protein IolR (Figure 6). The lack of conservation of several components of the *myo*-inositol network in *B. japonicum* decreases our confidence in the functional predictions presented for this species in Figure 6 relative to our predictions for *S. meliloti*, *M. loti*, *B. melitensis*, and *A. tumefaciens*. We propose that if *B. japonicum* can metabolize *myo*-inositol, it employs a different regulatory mechanism, and perhaps enzymatic strategy, than the other α-proteobacteria in our cross-species alignment.

### Experimental Validation of Cross-Species Functional Predictions in *Sinorhizobium meliloti*

The cross-species network predicted that the operon Smb20712-4 (NP_437959, NP_437960, NP_437961) in *S. meliloti* 1021 is a *myo*-inositol transporter (Figure 6A). This ABC transporter operon in *S. meliloti* 1021 is annotated in GenBank as a putative rhizopine transporter, based on homology to the known MocB rhizopine transporter in *S. meliloti* strain L5-30 [43]. While *S. meliloti* 1021 cannot metabolize rhizopine [3], rhizopine is derived from *myo*-inositol [43] suggesting that homology to MocB is a predictor of *myo*-inositol transport. However, a BLAST search of the *S. meliloti* 1021 genome using the sequence of *C. crescentus* IbpA inositol-binding protein as a query did not identify the periplasmic binding protein of the Smb20712-4 operon as the top hit, but rather another protein, Smb20072, that is also annotated as a periplasmic rhizopine-binding protein. Indeed, a simple BLAST search revealed several different ABC transporter operons in *S. meliloti* with high probability scores to the experimentally-defined *C. crescentus myo*-inositol transporter (see Table 3 for four candidate operons). Thus, simple pairwise comparisons with the known *myo*-inositol transporter of *C. crescentus* cannot easily distinguish the *myo*-inositol transport system in *S. meliloti* 1021. Instead, several additional search criteria must be imposed before Smb20712-4 is assigned the highest confidence score as the principal *myo*-inositol transporter. Specifically, while other operons in *S. meliloti* showed higher overall homology with select subunits of the *C. crescentus* ABC transporter, the operon Smb20712-4 clearly showed the highest conservation across all six species in our network alignment (Table 3), and the promoter region of this operon also contained the conserved palindromic motif first identified in *C. crescentus* (Figure 6B).

**Table 3:** Using the Graemlin network alignment algorithm [37], four candidate *myo*-inositol ABC transporter operons in *S. meliloti* were initially identified using the experimentally-defined *C. crescentus* inositol module as an alignment template.

Candidate operon in *S. meliloti*	*C. crescentus*	*A. tumefaciens*	*B. japonicum*	*B. melitensis*	*M. loti*
*SMb20712 (sugar binding protein)*	2.0e-22	**3.8e-129**	**1.1e-21**	**3.3e-34**	2.6e-23
*SMb20713 (ATP-binding protein)*	**7.1e-197**	**2.3e-246**	1.3e-108	**2e-117**	**8.3e-113**
*SMb20714 (permease protein)*	4.3e-34	**3.0e-122**	8.8e-29	7e-37	3.7e-38
SMb21345 (sugar binding protein)	**1.1e-26**	–	3.0e-16	4.4e-18	**7.0e-29**
SMb21344 (ATP-binding protein)	8.7e-113	7.0e-98	1.5e-104	8e-98	6.6e-102
SMb21343 (permease protein)	6.4e-30	3.7e-32	4.2e-31	2.3e-35	1.1e-34
SMb21377 (sugar binding protein)	5.5e-13	–	–	2.7e-6	–
SMb21376 (ATP-binding protein)	2.8e-103	2e-118	1e-103	1.4e-112	1.7e-105
SMb21375 (permease protein)	1.8e-48	7.5e-41	**1.1e-44**	2.5e-58	4.1e-37
SMb20856 (sugar binding protein)	2.6e-18	3.4e-13	1.6e-17	4.2e-11	4.9e-22
SMb20855 (ATP-binding protein)	1.3e-108	1.5e-114	**5.7e-112**	8.5e-92	2.4e-80
SMb20854 (permease protein)	**2.0e-52**	1.9e-29	1.7e-35	**3.4e-60**	**1.1e-50**

The BLAST score of each of the three protein subunits encoded in these candidate transporter operons was calculated against the equivalent protein subunits in each of our predicted *myo*-inositol ABC transporters from *Agrobacterium tumefaciens*, *Bradyrhizobium japonicum*, *Brucella melitensis*, and *Mesorhizobium loti*, and against the known *myo*-inositol ABC transporter of *C. crescentus*. For each species, the highest scoring hit is shown in bold. Instances in which the protein in our final network alignment was not contained within the top 20 BLAST scores are left blank. The *S. meliloti* operon predicted to function as a *myo*-inositol transporter in our final network alignment (see Figures 6 & 7) is highlighted in italics. Although this operon does not contain the highest scoring hits to each of the known subunits of the *C. crescentus* transporter, it has the highest overall conservation across the species included in our multiple network alignment (8 out of 15 possible top hits).

To experimentally test our prediction, we tested the growth of strains of *S. meliloti* Rm2011 (a direct derivative of *S. meliloti* 1021) carrying Tn5 transposon insertions in either *iolA* (NP_384832 ) or in the predicted ABC transporter periplasmic binding protein gene, Smb20712 [24], on GTS minimal medium [25] supplemented with either glucose or *myo*-inositol as the sole carbon source. Both Tn5 mutant strains grew normally in GTS-glucose and in Luria-Bertani (LB) medium. However, the *iolA*::Tn*5* and Smb20712::Tn*5* mutant strains did not grow on GTS with *myo*-inositol as a sole carbon source (Table 4). These results show that *S. meliloti* IolA is required for growth on *myo*-inositol and confirm our cross-species computational prediction that the protein Smb20712 is the functional homolog of the *C. crescentus* periplasmic inositol-binding protein IbpA. As such, we have annotated Smb20712 as *ibpA*, Smb20713 as *iatA*, and Smb20714 as *iatP*.

**Table 4:** Doubling times, in minutes, of *S. meliloti* strains grown in Luria-Bertani (LB) broth, and minimal GTS medium supplemented with either glucose (GTS-G) or *myo*-inositol (GTS-I) as the sole carbon source.

Strain	LB	GTS-glucose	GTS-inositol
Rm2011	198±2.9	223±8.7	226±3.3
Rm2011 *iolA*::Tn*5*	208±2.9	277±37.8	No growth
Rm2011 *ibpA*::Tn*5*	197±1.8	222±4.2	No growth

No growth indicates a lack of any visible cell growth after 1 week of shaken incubation.

### Conclusions

Using forward and reverse genetic strategies, we have defined genes in *C. crescentus* involved in the metabolism of the abundant environmental sugar, *myo*-inositol. These experiments uncovered an ABC *myo*-inositol transporter, and identified a novel *myo*-inositol regulatory gene and conserved *cis*-acting promoter regulatory sequence that control gene expression. Together, these genes and regulatory sequences form a metabolic module that ensures *C. crescentus* can regulate gene expression in response to *myo*-inositol, transport the sugar across its inner membrane, and catabolize the sugar to form the central metabolite acetyl-CoA. Expanding upon these traditional genetic studies, we also presented a schema for generating reliable cross-species annotation of an entire functional genetic module. Specifically, using statistical and computational methods we leveraged our experimental work on *C. crescentus myo*-inositol metabolism to produce high-quality gene annotations for functionally-homologous *myo*-inositol transporters, catabolic enzymes, and transcriptional regulators in five related α-proteobacteria. The *myo*-inositol genes in all of these species were noncontiguous on their respective chromosomes (Figure 7), making it difficult to predict function using co-location.

Our work has demonstrated the efficacy of combining a statistical protein network prediction algorithm [34] and the Graemlin network alignment algorithm [37] in the prediction and extrapolation of metabolic gene function. The method is significantly more robust than simple sequence comparison as a means to transfer annotation across species. Our network prediction and alignment protocol was validated on multiple levels: First, identification of the palindromic regulatory motif that was defined experimentally in *C. crescentus* (Figure 4) in the upstream regions of homologous genes/operons in our cross-species network alignment (Figure 6B & 7) provided excellent correlative validation of our alignment and refinement methodology. Second, we directly validated our cross-species functional prediction in *S. meliloti*, demonstrating that transposon disruption of the *iolA* gene and the Smb20712-Smb20714 transporter operon abolished growth on *myo*-inositol as the sole carbon source.

As the number of microbial genome sequences continues to grow, it is imperative that we develop improved methods to define and assign functions to genes, and reliably propagate this functional information across species. This study demonstrates that combining directed genetic, genomic and molecular experiments, statistical functional prediction, and global network alignment provides a powerful means to define and propagate gene function at the pathway level.

## Supporting Information

Table S1 Supporting data for DNA microarray experiments. Supporting information file contains two tables that report expression values for *Caulobacter* grown in glucose versus myo-inositol.
(0.09 MB DOC)
