# Supplementary material for: Genetic and Computational Identification of a Conserved Bacterial Metabolic Module
Source: PLoS Genet. 2008 Dec 19;4(12):e1000310. doi: 10.1371/journal.pgen.1000310 (PMC2597717; doi:10.1371/journal.pgen.1000310)
Supplement: Table S1 — Supporting data for DNA microarray experiments. Supporting information file contains two tables that report expression values for Caulobacter grown in glucose versus myo-inositol. (0.09 MB DOC) [file pgen.1000310.s001.doc]

Supplemental Tables. The full microarray dataset is publically available in the GEO database (<http://ncbi.nlm.nih.gov/geo>) under accession number GSE12414.

**Genes with at least two-fold greater expression in *myo*-inositol relative to glucose.**

| ORF | Gene | Annotation | I/G mRNA ratio |
| --- | --- | --- | --- |
| CC1764 | *aceA* | isocitrate lyase | 4.45 |
| CC1314 |  | ABC transporter, HlyB/MsbA family | 4.03 |
| CC1298 | *iolC* | iolC protein | 4.01 |
| CC1299 | *iolD* | iolD protein | 3.86 |
| CC1302 | *iolA* | malonate-semialdehyde dehydrogenase, putative | 3.82 |
| CC2765 |  | sensor histidine kinase | 3.58 |
| CC1683 |  | sensor histidine kinase, putative | 3.52 |
| CC1171 |  | 2-hydroxychromene-2-carboxylate dehydrogenase, putative | 3.26 |
| CC0859 | *ibpA* | sugar ABC transporter, periplasmic sugar-binding protein | 3.18 |
| CC1300 | *iolE* | iolE protein | 3.10 |
| CC1296 | *idhA* | myo-inositol 2-dehydrogenase | 3.01 |
| CC2527 |  | transcriptional regulator, AraC family | 2.86 |
| CC3028 |  | transcriptional regulator, ArsR family | 2.61 |
| CC2258 |  | heat shock protein, Hsp20 family | 2.47 |
| CC1304 |  | DNA-binding response regulator | 2.45 |
| CC2392 |  | transcriptional regulator, GntR family | 2.41 |
| CC2029 | *thiC* | thiamine biosynthesis protein ThiC | 2.38 |
| CC1840 |  | ribonuclease BN, putative | 2.32 |
| CC2766 |  | DNA-binding response regulator | 2.32 |
| CC0267 | *dnaX* | DNA polymerase III, gamma and tau subunits | 2.18 |
| CC0081 |  | transcriptional regulator, putative | 2.13 |
| CC0716 |  | L-lysine 2,3-aminomutase, putative | 2.13 |
| CC0147 |  | glutathione S-transferase family protein | 2.09 |
| CC3147 |  | TonB-dependent receptor | 2.06 |
| CC2809 |  | peptidase, M20/M25/M40 family | 2.05 |
| CC2232 |  | transcriptional regulator, Cro/CI family | 2.05 |
| CC2884 |  | sensor histidine kinase | 2.01 |

The following hypothetical proteins also showed at least 2-fold upregulation in myo-inositol: CC0083, CC0660, CC841, CC854, CC1325, CC1413, CC1613, CC2233, CC2435, CC2700, CC2764, CC3027, CC3073, CC3074, CC3079, CC3184, CC3220, CC3263, CC3444, CC3458, CC3554, CC3647 and CC3705.

**Genes with at least 2-fold or greater expression in glucose relative to *myo*-inositol.**

| ORF | Gene | Annotation | G/I mRNA ratio |
| --- | --- | --- | --- |
| CC1106 |  | conserved hypothetical protein | 19.41 |
| CC0797 | *celD* | 1,4-beta-D-glucan glucohydrolase D | 13.15 |
| CC1517 |  | TonB-dependent receptor | 10.65 |
| CC0214 |  | TonB-dependent receptor | 6.56 |
| CC1518 |  | ABC transporter, ATP-binding protein | 6.09 |
| CC1519 |  | hypothetical protein | 6.00 |
| CC2056 | *pgl* | 6-phospho-glucono-lactonase | 5.31 |
| CC2055 | *edd* | phosphogluconate dehydratase | 5.09 |
| CC0556 |  | hypothetical protein | 4.36 |
| CC0559 |  | hypothetical protein | 3.55 |
| CC0798 |  | sialic acid-specific 9-O-acetylesterase, putative | 3.46 |
| CC2564 |  | transcriptional regulator, LysR family | 3.32 |
| CC0557 |  | hypothetical protein | 3.23 |
| CC2054 | *glk* | glucokinase | 3.12 |
| CC1520 |  | hypothetical protein | 3.11 |
| CC2053 |  | transcriptional regulator, LacI family | 2.89 |
| CC2047 |  | conserved hypothetical protein | 2.76 |
| CC0554 |  | hypothetical protein | 2.63 |
| CC2745 |  | hypothetical protein | 2.58 |
| CC1406 |  | ferredoxin, putative | 2.47 |
| CC0814 |  | major facilitator family transporter | 2.40 |
| CC0320 |  | hypothetical protein | 2.33 |
| CC2051 | *pyk* | pyruvate kinase | 2.22 |
| CC0896 |  | GGDEF family protein | 2.22 |
| CC1630 |  | oxidoreductase, Gfo/Idh/MocA family | 2.21 |
| CC0482 | *metE* | 5-methyltetrahydropteroyltriglutamate-homocysteine methyltransferase | 2.09 |
| CC3103 | *purA* | adenylosuccinate synthetase | 2.04 |
